# RACIAL AND ETHNIC DIFFERENCES BETWEEN GRIP STRENGTH AND FUNCTIONAL LIMITATIONS: RESULTS FROM NHATS 2010-2014

**DOI:** 10.1093/geroni/igz038.2520

**Published:** 2019-11-08

**Authors:** Elizabeth Vasquez, Ana R Quiñones, Lenore Gensburg

**Affiliations:** 1 University at Albany, SUNY, Albany, New York, United States; 2 Family Medicine, Oregon Health & Science University, Portland, Oregon, United States; 3 University at Albany, New York, United States

## Abstract

Current research regarding grip strength highlights the robustness of grip strength as a predictor of morbidity and mortality. The aim of this study was to evaluate the association of grip strength over four years with functional limitations among racially/ethnically diverse older adults. We analyzed National Health and Aging Trends Study (NHATS) data 2010-2014. Our sample included 4,413 adults > 65 years old. Functional limitation was defined as a sum of difficulty performing eight ADL/IADLs (range 0-8) at each wave. Grip strength was measured using a digital hand dynamometer and readings were recorded in kilograms (kg) (maximum of 32 kg for men and > 20 kg for women). We estimated stratified linear regression models by race/ethnicity and age, and adjusted for BMI, education, and gender. The majority of the sample was between 65-79 years of age (64%), 55.1% were female and the average BMI was 27.5. We found that differences in ADL/IADL limitations increased and grip strength decreased over the four year period of observation. We also found racial/ethnic differences between waves 1 and 4 with greater ADL/IADL limitations for Hispanics with lower grip strength scores compared to non-Hispanic whites. There were racial/ethnic differences in the association between grip strength and ADL/IADL over time in Non-Hispanic blacks and Hispanics when compared to Non-Hispanic whites. This is an important issue to address since loss of muscle strength in older adults may lead to several negative outcomes such as limited activities of daily living which may affect older adults differentially based on race/ethnicity.

